# In-situ analysis of sub-nanomolar level of Fe(II) in open-ocean waters

**DOI:** 10.1007/s44211-024-00637-0

**Published:** 2024-07-30

**Authors:** Hajime Obata, Akira Mase, Toshitaka Gamo, Jun Nishioka, Kei Okamura

**Affiliations:** 1 Atmosphere and Ocean Research Institute, The University of Tokyo, 5-1-5 Kashiwanoha, Kashiwa, Chiba 277-8564 Japan; 2 Low Temperature Research Institute, Hokkaido University, Hokkaido, Japan; 3 Research and Education Faculty, Kochi University, Kochi, Japan

**Keywords:** Luminol chemiluminescence, Seawater, Iron, GEOTRACES, Indian Ocean

## Abstract

Iron (Fe) in seawater is an essential micronutrient for marine phytoplankton, and Fe deficiency limits their growth in high-nutrient, low-chlorophyll areas. The bioavailability of Fe for phytoplankton largely depends on its chemical speciation in seawater. In surface water, the reduction of Fe(III) to Fe(II) is an important step in the uptake of Fe by phytoplankton. However, the marine biogeochemical cycle of Fe(II) in the open ocean has not been fully investigated. In oxic open-ocean waters, Fe(II) is rapidly oxidized and exists at sub-nanomolar levels, making it difficult to determine the Fe(II) concentration of seawater. In this study, we applied the flow analytical method of determining the Fe(II) concentration of seawater using luminol chemiluminescence in an in-situ analyzer (geochemical anomaly monitoring system, GAMOS). In the onboard laboratory, we successfully detected sub-nanomolar levels of Fe(II) in seawater using the GAMOS. In the central Indian Ocean, this analyzer was deployed at a depth of 1000 m to determine the Fe(II) concentration in the water column. During deployment, the detection limit (0.48 nM) was insufficient to determine the concentration. Therefore, we need to lower the blank values and enhance the stability of signal of the in-situ analytical method for application to open-ocean seawater samples.

**Graphical abstract:**

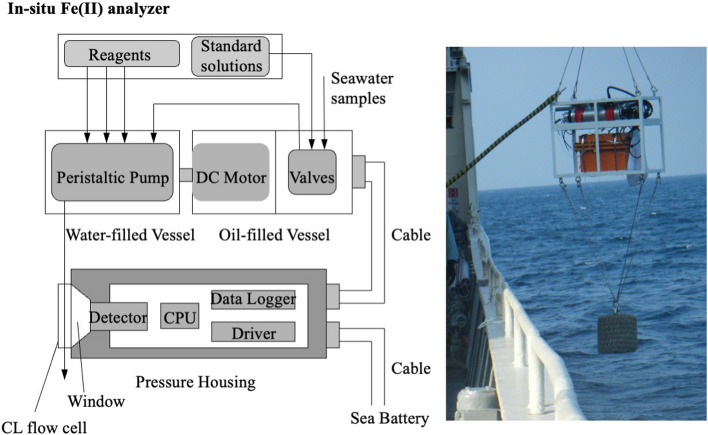

## Introduction

Iron (Fe) is an essential micronutrient for marine phytoplankton and is depleted in the surface waters of high-nutrient, low-chlorophyll (HNLC) areas. In 30% of the ocean surface, Fe deficiency limits phytoplankton growth [1]. To understand the bioavailability of Fe to phytoplankton, both the Fe concentration and its speciation in seawater are important [2]. In seawater, Fe(III) is thermodynamically stable under oxic conditions [3]. It is also known that more than 99% of dissolved Fe(III) is organically complexed [4]. In HNLC areas, however, picomolar levels of Fe(II) have been detected in the Southern Ocean [5–7] and the subarctic Pacific [8–10]. Some model studies have indicated that the reduction of Fe(III) to Fe(II) with subsequent re-oxidation to Fe(III) is an important process for Fe acquisition by phytoplankton [11–13], implying that Fe(II) is more bioavailable to phytoplankton than Fe(III). Hence, Fe(II) plays a key role in Fe chemistry and bioavailability in the ocean.

Several methods have been developed to determine Fe(II) levels in seawater. Using chelating resin column preconcentration, sub-nanomolar amounts of Fe(II) have been determined using chemiluminescence or catalytic spectrophotometric methods [5, 14–16]. Without column preconcentration, adsorptive cathodic stripping voltammetry has been applied for determining the Fe(II) concentration of seawater [17, 18]. In this method, the Fe(III) concentration was determined by adding 2,2-bipyridyl and masking Fe(II), and then the Fe(II) concentration was calculated as the difference in total Fe concentration. This approach is unsuitable for seawater samples with considerably low Fe concentrations. Because Fe(II) is rapidly oxidized to Fe(III) under oxic conditions, it is preferable to analyze Fe(II) in seawater immediately after sampling. The half-life of Fe(II) in seawater was estimated to be 3.2 min at 25 °C and pH 8 [19]. In cold surfaces or suboxic waters, the half-life is on the order of hours to days [8, 20–22].

A rapid, direct analytical method that has long been used to determine the Fe(II) concentration of water samples is a colorimetric method, where a specific ligand such as ferrozine (3-(2- pyridyl)-5,6-bis(4-phenylsulfonic acid)-1,2,4-triazine]) is employed [23]. By using a long liquid waveguide capillary flow cell, trace levels of Fe(II) were determined (e.g., ferrozine [24, 25] and ferene (3-(2-pyridyl)-5,6-di(2-furyl)-1,2,4-triazine-5′,5″-disulfonic acid disodium) [26]). Recently, catalytic spectrophotometry using *N*,*N*-dimethyl-*p*-phenylenediamine dihydrochloride (DPD) was applied for Fe(II) determination in seawater [27]. The DPD did not react specifically with Fe(II). In this method, Fe(III) was selectively removed by acidifying the sample and passing it through a chelating resin column, which enabled the direct detection of Fe(II). For the direct analysis of Fe(II) in seawater, the luminol chemiluminescence method is most commonly used [28]. Based on the catalytic effect of Fe(II) on the oxidation of luminol, which luminesces, a sub-nanomolar amount of Fe(II) was successfully detected in seawater [29]. As the detection limit can reach picomolar levels, this method is applicable for the determination of Fe(II) in open ocean waters ([6–10, 22, 30–36]. To avoid rapid oxidation, seawater samples are often acidified [33, 37] to stabilize Fe(II) for tens of minutes. However, acidification sometimes unintentionally reduces Fe(III) in seawater [37]. Therefore, the best way to rapidly determine Fe(II) in seawater is in situ [38, 39].

For chemical studies in hydrothermal environments, in-situ analyzers have been developed to determine Fe concentration using ferrozine [40–43]. Among these surveys of hydrothermal activity, micromolar levels of Fe(II) were detected by Alchimist at the Rainbow hydrothermal vent field [42]. Later, an in-situ automatic analyzer (METIS) for Fe(II) was developed using ferene [26]. The analyzer was used to obtain the vertical profile of Fe(II) in the Baltic Sea (< 200 m depth). An autonomous spectrometric analyzer for Fe(II) with ferrozine and Mn with 1-(2-pyridylazo)-2-naphthol (PAN) in seawater was developed using a microfluidic method [44] that was applied to the water column of the Baltic Sea (< 300 m depth). The analyzer was also used in an estuarine system with highly dissolved organic matter [45]. Another autonomous lab-on-a-chip analyzer using ferrozine was also developed for the determination of Fe(II) and labile Fe in coastal waters [46] and was used for time-series deployment in shallow waters in the Kiel Fjord. All analyzers that use the colorimetric method offer nanomolar detection limits, which are difficult to adopt in open-ocean waters.

In this study, a flow analytical method for determining the Fe(II) concentration of seawater using luminol chemiluminescence was applied to an in-situ analyzer (geochemical anomaly monitoring system, GAMOS), which was originally developed for manganese (Mn) determination in the study of hydrothermal plumes [47]. For determination of nanomolar levels of manganese in hydrothermal plumes, a flow through technique using luminol-hydrogen peroxide chemiluminescence detection had been introduced to the GAMOS system. The Mn analyzer was previously used to trace hydrothermal activities in deep oceanic layers (~ 3600 m depth) [47–51]. We conducted the first trial of the GAMOS system for the determination of sub-nanomolar levels of Fe(II) in open-ocean waters.

## Experimental section

### Reagents

All solutions were prepared using purified water from a Millipore system (Milli-Q water, MQW). To clean the low-density polyethylene (LDPE) bottles, surfactants (Extran MA01, Merck) and hydrochloric acid (HCl; Guaranteed Reagent, Wako Chemicals) were used. Ultrapure-grade aqueous ammonia, HCl (Tamapure AA-100, Tama Chemicals), and luminol sodium salt (Sigma-Aldrich) were purchased commercially. We dissolved 0.737 g of luminol sodium salts in 250 mL of MQW to prepare a 14.8 mM luminol solution. The solution containing luminol (0.74 mM), aqueous ammonia, HCl, and MQW was heated to 60 °C for over 15 h [37] and left in the dark until the temperature reached room temperature. Standard stock solutions of Fe(II) (10 mM) were prepared by dissolving ferrous ammonium sulfate hexahydrate (Wako Chemicals) in a 0.1 M HCl solution before the research cruise and stored in a refrigerator until use. Standard working solutions (1 µM) were prepared by diluting the stock solutions with 0.1 M HCl before each use. All solutions were prepared in LDPE bottles (Nalge).

### Flow analytical system

**Fig. 1:**
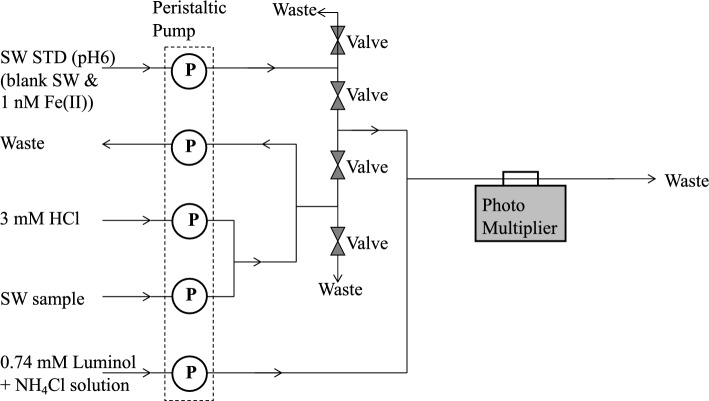
Flow diagram of the analytical system. P: peristaltic pump, SW: seawater

Highly sensitive luminol chemiluminescence detection, previously used for onboard determination of Fe(II) in oceanic waters [29, 30, 36, 37], was applied to the flow analytical system (Fig. 1). In this system, unfiltered seawater samples were mixed with a luminol reagent under alkaline conditions (pH 10.2). The system comprised a five-channel peristaltic pump (Masterflex, 7013-20), a two-way solenoid Teflon valve (Takasago), and a chemiluminescence (CL) detector (Fig. 1). A five-channel peristaltic pump was used to deliver the sample solution, diluted HCl (0.003 M), standard solution, and luminol solution mixed with aqueous ammonia and ammonium chloride. Pharmed tubes (Norton) were used as peristaltic pumps. Polyethylene bags were used to store the reagents during the deployment of the GAMOS. The standard solution was prepared by mixing aged seawater with HCl. The pH was adjusted to 6 prior to use. The sample solution was mixed with diluted HCl in situ within the flow system, and the pH of the mixture was adjusted to 6. The seawater pH could be adjusted to 6.0 by mixing with hydrochloric acid as indicated in the previous study [37].

The rotation rate of the motor for the peristaltic pump was fixed at 10 rpm owing to mechanical limitations. We used Pharmed tubes with varying IDs (1.6, 0.8, and 3.1 mm) for the peristaltic pump, which resulted the flow rates of 3.8 mL/min for the seawater sample, 1.0 mL/min for HCl and 13.4 mL/min for luminol solution. Because the flow rate of the seawater sample and HCl mixture (4.8 mL/min) was higher than that of the standard solution (3.8 mL/min), a fraction was removed from the system (1.0 mL/min) using a peristaltic pump (Fig. 1). The flows of the seawater samples and standard solution were switched using two-way solenoid valves. All flow lines were 1 mm inner diameter (ID) Teflon tubes, which were connected to Teflon connectors [52]. The seawater sample or standard solution was immediately mixed with the luminol solution. The mixture was introduced into the flow cell of the CL detector.

As previously described [38], the reliability over time should be examined to test the method. To ensure the stability of the baseline, the flow line between the blank seawater and seawater samples was switched every 2 min. By switching the flow lines frequently, we confirmed that no drift occurred during the deployment.

### Cleaning method

The Teflon tubes and fittings were cleaned using the method adapted for the analysis of Fe in seawater [52, 53]. LDPE bottles were soaked in 5% surfactant (Extran MA01, Merck) for 1 d and then 3 M HCl for 1 d. These bottles were filled with 0.1 M HCl and then heated in a microwave oven at 60–70 °C and left overnight at room temperature. The same procedure was repeated using MQW. Polyethylene bags for the reagents were filled with 0.1 M HCl overnight, and then MQW overnight. Pharmed tubes for the peristaltic pump were filled with 1% surfactant (Extran MA02, Merck), 0.1 M HCl, and MQW for 1 d, respectively. Teflon tubes in the flow system were filled with 0.1 M HCl and MQW for 1 d each.

### Instruments

**Fig. 2:**
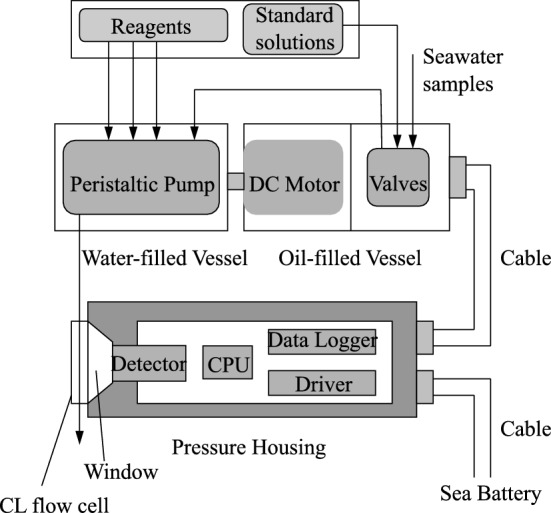
Composition of the GAMOS for Fe(II) analysis

In this study, a direct Fe(II) determination method was applied to a GAMOS system [47]. The in-situ analyzer was composed of an acrylic, oil-, and water-filled pressure-compensated vessel containing a flow-through analyzing system, an aluminum pressure housing for the electronic modules, and a battery for the power supply (Fig. 2). In the acrylic water-filled vessel, a peristaltic pump with five cartridges, Pharmed tubes, and Teflon tubes were stowed. The motor and two-way solenoid Teflon valves were placed in an oil-filled vessel. The vessels were equipped with rubber diaphragms on their tops for pressure balance between the inside and outside of the vessels because small air bubbles often remained inside. The photomultiplier detector with amplifiers as a CL detector, central processing unit (CPU), and data logger were placed in the aluminum pressure housing. A stepping motor is used to rotate the peristaltic pump. The CL flow cell was 1 mm ID Teflon tubing coiled into a 20 mm diameter groove on an aluminum block.

The aluminum pressure housing, whose pressure resistance was equivalent to a depth of 5200 m, stored a CPU for system control, a photomultiplier detector with amplifiers, an eight-channel 10-bit ArD converter, and a 2 Mb flash memory for data logging. The pressure housing has an acrylic window at the top for CL detection (Fig. 2). The CL flow cell was attached to an acrylic window outside the pressure housing, and the aluminum housing was connected to the acrylic vessel using underwater cables (Brantner and Associates, RMK-8-FS) and connectors (Brantner and Associates, XSK-8). The detector voltages were amplified (× 1, × 100, and × 1000), digitized, and stored at preprogrammed intervals. Data can also be collected after deployment and displayed on a computer on the ship. Electric power was supplied by SeaBattery Power Modules (SB-24/40, DeepSea) at a voltage of 24 V.

### Field observation

**Fig. 3:**
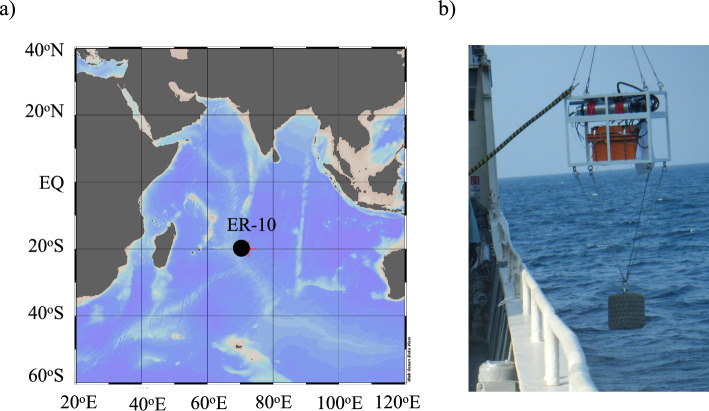
**a** Sampling station (ER-10) during R. V. Hakuho-maru KH-09-5 cruise. **b** Photo of GAMOS deployment

We performed an in-situ analysis of Fe(II) in seawater at Stn. ER-10 during the R. V. Hakuho-maru KH-09-5 cruise in the Indian Ocean (Fig. 3a), a GEOTRACES transect cruise (GI04) led by the Japanese GEOTRACES group. During the cruise, we deployed the GAMOS system several times with a weight of 100 kg using a titanium wire with the No. 3 winch of R. V. Hakuho-maru (Fig. 3b), which was applicable for clean sampling [54]. The measurements were performed at a winch speed of 1 m s^–1^ when lowering and pulling the GAMOS. Discrete samples were also collected using Niskin-X samplers deployed on a CTD-carousel multiple sampling system (CMS) connected to a titanium armored cable [55]. We compared the data obtained using the GAMOS with those obtained by discrete sampling at the same station.

### Onboard measurement

Using an onboard analytical method, we determined the dissolved Fe(II) in seawater samples collected discretely during the KH-09-5 cruise. The details of this analytical method have been described previously [36]. The seawater samples were filtered through a 0.2 µm pore-size capsule filter (Acropak, Pall) by gravity within a clean area in the R.V. Hakuho-maru and filled into LDPE bottles. Immediately after sampling, the seawater samples were acidified with HCl to pH 6 as indicated in the previous study [37]. The acidified samples were measured within 15 min of acidification. The detection limit was 0.012 nM, which was triple the standard deviation of low-concentration standard solution measurements.

## Results and discussion

### Calibration in the laboratory during the cruise

**Fig. 4:**
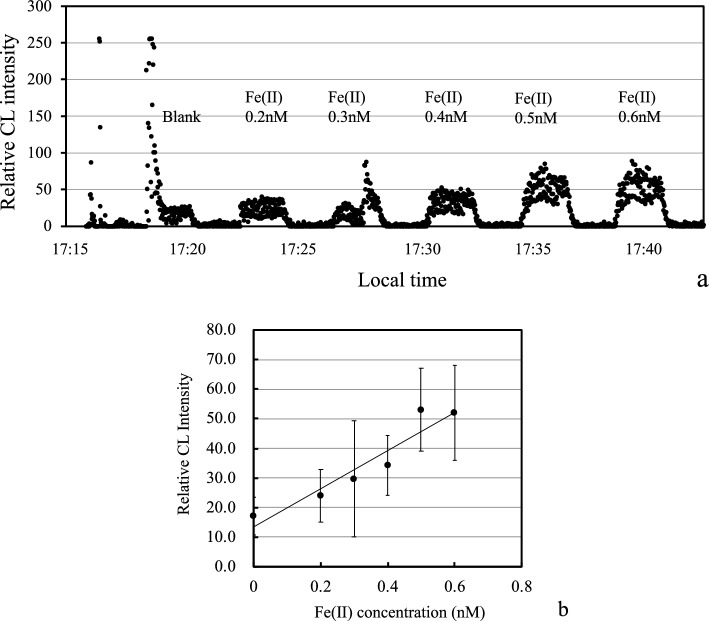
**a** Time variation of chemiluminescence intensities during calibration in the onboard laboratory. **b**) Calibration curve generated by the GAMOS system. After the CL intensity reached a plateau in (**a**), continuous data for 72 s were used for the calibrations. The error bars indicate the standard deviation of the CL intensities during the measurements

During the research cruise, we tested the GAMOS using a luminol CL detection system for Fe(II) in seawater at an onboard laboratory. We prepared seawater standard solutions containing 0.2, 0.3, 0.4, 0.5, and 0.6 nM Fe(II). The continuous CL intensity data for the calibration are shown in Fig. 4a. The high CL intensities at 17:19 were caused by accidental air introduction to the sampling line. The blank values were relatively high because the sampling line was slightly contaminated. The CL intensity increased linearly with the addition of Fe(II). The calibration curve is shown in Fig. 4b. We obtained a linear relationship between the added Fe(II) concentration (R^2^ = 0.9013, *p* < 0.05) and the CL intensities, though large errors were observed in this low-concentration range probably because the slow rotation rate of the peristaltic pump caused the fluctuation of the CL intensities. The detection limit was 0.074 nM, which was triple the standard deviation of the baseline CL intensity.

### Stability of the CL signals

**Fig. 5:**
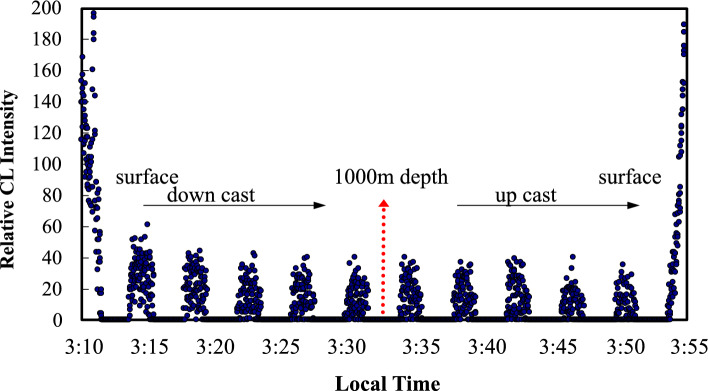
Time variation of chemiluminescence intensities for the seawater sample containing 1 nM Fe(II) during the deployment of the GAMOS from the surface to a 1000 m depth

**Fig. 6:**
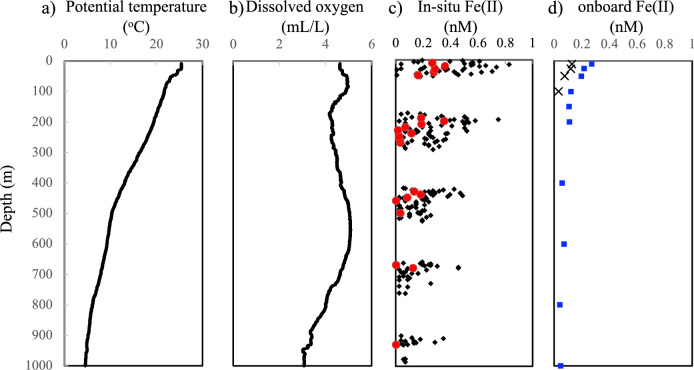
Vertical profiles of **a** potential temperature, **b** dissolved oxygen by a sensor, **c** Fe(II) concentrations calculated from the CL intensities by GAMOS (black closed circles: continuous data; red closed circles: averaged values at 10 m depth interval), and **d** onboard measurements of dissolved Fe(II) (blue closed circles: this study; crosses: data from Kondo and Moffett [33])

We investigated the in-situ stability of the CL intensity depending on Fe(II) addition during GAMOS deployment in the ocean. The carrier seawater containing 1 nM Fe(II) was prepared at pH 6 (Fig. 1). The GAMOS system was deployed at a 1000 m depth. At the beginning and end of the measurements, the CL intensity changed rapidly owing to the intrusion of ambient sunlight on the deck. During 42 min of deployment, the CL intensity decreased slightly (≈ 9%) with time (Fig. 5). Considering the variability of the signals (Fig. 4b), this change was insignificant. Figure 6a shows the vertical profile of the water temperature, which decreased from 25.4 °C at the surface to 4.6 °C at a 1000 m depth. The pressure also increased up to a depth of 1000 m. Although the temperature increased and pressure decreased, the CL intensity change was within the standard deviation of the CL baseline intensities. In the Mn analyzer, temperature affected the CL intensity [47]. These different trends may be caused by the different CL mechanisms. In the Mn analyzer, the luminol–hydrogen peroxide system is mediated by a manganese–triethylenetetramine complex. In this study, luminol CL was caused by reactive oxygen species produced by Fe(II) [29], where the mechanism may depend on the temperature. In our study, the reagents were stored in the refrigerator immediately before deployment, which might have minimized the temperature dependence. The small variation may also correspond to slow Fe(II) oxidation at pH 6 in open-ocean water, as indicated previously [36, 37].

### Vertical profile of Fe(II) in seawater

At Station ER-10, the GAMOS system was deployed at a depth of 1000 m. Calibration was performed before deployment. The system was hung with a titanium cable with a depth of up to 1000 m at a winch speed of 1 m/s. To avoid the sunlight intrusion in the surface layer, the in-situ measurement was carried out after sunset. The obtained data are shown in Fig. 6c along with the dissolved oxygen data.

The variation in CL intensities was relatively large; therefore, the detection limit estimated from the continuously obtained data was ≈ 0.48 nM (three times the standard deviation of the baseline CL intensity). The detection limit was much higher than that obtained using the GAMOS system in the onboard laboratory (0.07 nM), probably owing to the flow fluctuations caused by the peristaltic pump. Because the CL intensity rapidly decreased over time in this system, the mixture of seawater samples and luminol solution was quickly transferred to the flow cell. The fluctuation of flows by the peristaltic pump can induce variations in the arrival time of the flow cell and variable CL intensities. At low temperatures, the tubes of the peristaltic pump became harder, which may have caused a large fluctuation in the flow rates of the peristaltic pump.

To cancel the effect of the fluctuation, we averaged the data for 10 m intervals, as indicated in Fig. 6c. Using the 10-m averaged data below the euphotic zone (210–930 m depth), the detection limit was calculated as 0.19 nM. Most data below 930 m depth were less than detection limit, thus only one data was indicated as a red circle at the bottom of Fig. 6c. The Fe(II) concentrations were high (< 0.19–0.36 nM) in surface waters, decreasing as the depth increased (0–200 m depth). The detection limit of the in-situ analyzer from the 10-m averaged data was too high yet for application to open-ocean seawater.

We also determined the dissolved Fe(II) concentrations of seawater samples collected using discrete water sampling and onboard analytical methods; the results are shown in Fig. 6d. Similar to the in-situ measurements, the Fe(II) concentration decreased as the depth increased, although the surface concentration was lower (0.27 nM) in the onboard measurements. Above 200 m depth, the averaged concentration by in-situ analysis (0.27 nM) was higher than that by the onboard analysis (0.17 nM). For the discrete samples, more than 10 min were required to recover the samplers and analyze the seawater. If the Fe(II) were oxidized for 10 min by following the previously reported oxidation rate in the Kuroshio region [36], the Fe(II) concentration would be less than 1% of the initial value. The difference between the in-situ analyses and the data obtained from the discrete samples was not so large that the rapid oxidation had not occurred during the sampling process. During the same research cruise, dissolved Fe(II) concentrations were previously reported [33], as indicated in Fig. 6d. Although the trend was identical, the reported concentrations were lower than our in-situ and onboard measurements. In their analytical method, 3-morpholinopropanesulfonic acid buffer was added to the seawater samples to adjust the pH to 7, while we added HCl to adjust the pH of the samples to 6. To compare the data obtained by different methods, more intercomparison efforts are required, as previously indicated [28].

Through in-situ measurements, we found that the Fe(II) concentration was higher than the Fe(III) concentration at the surface down to a 200 m depth at this station [55]. Owing to the high detection limit of the in-situ analysis, we may have obtained higher dissolved Fe concentrations than the actual concentrations. Moreover, the seawater samples were not filtered in the in-situ analyzer, while the samples for the dissolved Fe measurements were filtered using 0.2 µm pore-size filters. Labile particulate Fe(II) might exist in the euphotic zone as Fe(II) adsorbed onto biological surface, which can be detected by the luminol CL detection method as previously suggested [56]. Additionally, some interference in shallow waters may have affected the Fe(II) measurements. Any interference from the luminol CL detection methods, such as V(IV) [32] and organic compounds [57], should be considered. To reveal the biogeochemical cycles of Fe(II) in shallow waters, we need to improve in-situ analytical methods, particularly by lowering the in-situ detection limit.

## Conclusion

We tested an in-situ analyzer, GAMOS, for the determination of Fe(II) in open-ocean waters using the luminol CL method. In the onboard laboratory, we successfully drew a calibration curve for sub-nanomolar levels of Fe(II) in seawater using the GAMOS. The analyzer was deployed at a depth of 1000 m at a station in the central Indian Ocean to detect Fe(II) in the water column. During deployment, the detection limit (0.48 nM) was worse than that at the onboard laboratory and was insufficient to determine the Fe(II) concentrations in open-ocean waters. By analyzing discrete samples using the onboard analytical method, we found that the Fe(II) concentration (0.27 nM) in the surface water decreased with depth. This trend is similar to that observed in the in-situ analysis. To reveal Fe(II) distributions in the open ocean, we need to improve the in-situ analytical method by lowering the blank values and enhancing the signal stability.

## Data Availability

All data generated or analyzed during this study are included in this published article.
